# 2D speckle-tracking echocardiography as a prognostic imaging modality for COVID-19 adverse outcomes

**DOI:** 10.2217/fca-2022-0057

**Published:** 2022-11-02

**Authors:** Ehsan Goudarzi, Fateme Yousefimoghaddam, Alireza Ramandi, Isa Khaheshi

**Affiliations:** ^1^Cardiovascular Research Center, Shahid Beheshti University of Medical Sciences, Tehran, Iran; ^2^School of Medicine, Tehran University of Medical Sciences, Tehran, Iran; ^3^Students' Scientific Research Center (SSRC), Tehran University of Medical Sciences, Tehran, Iran

**Keywords:** adverse outcomes, COVID-19, hospitalization, intubation, left atrium, left ventricle, prognosis, right atrium, right ventricle, speckle-tracking echocardiography

## Abstract

**Aim:** 2D speckle-tracking echocardiography (2D-STE) has been used to assess cardiac recovery during the COVID-19 patient follow-ups within the pandemic. The novel role of STE in predicting adverse outcomes of COVID-19 has received attention due to its high sensitivity in identifying subclinical myocardial dysfunction. We reviewed the studies on using 2D-STE to assess COVID-19 prognosis. **Methods:** a literature search was conducted on PubMed and Scopus for eligible articles, 24 of which discussed using prognostic 2D-STE for COVID-19 patients. **Results:** 2D-STE predicts cardiovascular impairments more rapidly and precisely than conventional echocardiography. The 2D-STE technique presents an independent prognostic factor in COVID-19 infection. **Conclusion:** 2D-STE could be considered a time-efficient and accurate risk predictor of all-cause mortality in COVID-19 patients.

## Plain language summary

In this review, we have gathered every article that discusses the association between COVID-19 prognosis and speckle-tracking echocardiography, which is a novel, fast and accurate method and does not need expert operators to perform. We have shown that according to the current literature, we can use this imaging technique on the right and left heart ventricles to estimate the prognosis of the patients infected with COVID-19.

Initially known as a respiratory tract infection, coronavirus disease 2019 (COVID-19) is a multi-organ inflammatory disease that has led to high mortality and morbidity rates worldwide [1,2]. Several documents have addressed cardiorespiratory involvement as the most significant predictor of mortality in patients with COVID-19 infection [3]. Myocardial damage and heart failure are more incident in patients with severe COVID-19 infection [2]. Henceforth, early evaluation of lungs and heart function might prompt better disease outcomes.

Cardiovascular involvement in COVID-19 infection may be due to direct myocardial injury, increased systemic inflammatory response, hypoxia or micro-thrombogenesis resulting from a hypercoagulable state [4,5]. Several previous articles have indicated that the multi-systemic inflammation that occurs in COVID-19 infection may lead to multi-organ damage [4,5]. The other proposed mechanism of heart failure is increased right ventricular afterload due to severe respiratory infection [6]. Hence, assessing cardiac structure and function can provide helpful information regarding COVID-19 mortality. Since conventional Two-dimensional echocardiography _the most common modality of use lacks information about the intrinsic properties of the myocardium, some other techniques are being used to compensate for shortcomings. Speckle-tracking echocardiography (STE) can identify myocardial dysfunction and subclinical myocardial impairment [7]. STE also detects left ventricular (LV) dysfunction more sensitively than conventional echocardiography [8–10].

We herein aim to examine the role of 2D-STE as novel method in predicting adverse outcomes in COVID-19 patients. We have reported the prognostic value of 2D-STE imaging for each cardiac chamber individually.

## Methods

A review of the scientific literature was performed to investigate available articles focusing on the application of STE in COVID-19 patients as an imaging predicting parameter. A comprehensive literature search was performed on PubMed and Scopus to identify suitable studies that were published until March 2022. Our search strategy is as follows: Initially, a manual search was applied in PubMed. The resulting articles were used to obtain the list of keywords and Medical Subject Headings (mesh) terms. The search strategy was repeated multiple times in PubMed and Scopus search engines until the keywords list was completed and no further studies were found. Subsequently, a manual search of the bibliography was performed to find other potentially eligible studies. Search results were screened by title and abstract, and potentially eligible studies were further investigated based on full text. Throughout the review, no language restriction was applied. Data extraction was performed using the double data extraction method by two independent reviewers (FY, EG), followed by a scrutinous review of both included and excluded articles by two other members of the team (IK, AR).

## Results

We included 24 articles in our study, of which 20 were original, and the remaining 4 were either editorial, letters to the editor, or short communication. (Table 1) The sample size of the studies ranged from 9 to 428 patients. All patients had laboratory-confirmed COVID-19 infection as the inclusion criteria in each original research reviewed. While all of the included articles used 2D-STE, the studies differ in the specific type of strains measured, as shown in Table 2. The most common outcome under investigation was the association between strain measures and mortality.

**Table 1.** Association of adverse outcomes with different strains.

Strain (number of article	Associated with (number of articles)	Not associated with (number of articles)
**LV**		
GLS/LS (15)	Mortality (9) Intubation (1) Severity (1) Hospitalization (1) ARDS (1)	Mortality (4) Intubation (1)
GCS (2)	–	Mortality (1) Severity (1)
FWLS (1)	Intubation (1)	Mortality (1)
BLS (1)	–	Mortality (1) ICU admission (1) Intubation (1)
**RV**		
RV-LS/GS/LS (12)	Mortality (5) Intubation (2) ICU admission (1) Hospitalization (1)	Mortality (4) Intubation (2) Severity (1)
FWS/FWLS/FWGLS (9)	Mortality (6) Severity (2) Intubation (1)	Mortality (2) Severity (1) Intubation (1)
TWGLS (1)	–	Severity (1)
**LA**		
LAS/RA-LS (2)	Atrial fibrillation (1)	Mortality (1)
**RA**		
RA-LS (1)	–	Mortality (1)

ARDS: Acute respiratory distress syndrome; BLS: Basal longitudinal strain; FWLS: Free wall longitudinal strain; FWS: Free wall strain; FWGLS: Free wall global longitudinal strain; GS: Global strain; GLS: Global longitudinal strain; GCS: Global circumferential strain; LV: Left ventricle; LA: Left atrium; LS: Longitudinal strain; LAS: Left atrial strain; RV: Right ventricle; RA: Right atrium; TWGLS: Total wall global longitudinal strain.

**Table 2.** Review of literatures on 2D-STE and COVID-19 adverse outcomes.

Study	Sample size	Age (years), mean ± SD/median	Male sex (%)	Strain	Comorbidities (%)				Decreased strain associated with:		Ref.
					HF	CAD/ IHD	COPD/ Asthma	CKD	All-cause mortality	Other adverse outcome	
Lassen *et al.*	428	69	54.7	LVGLS RVLS	10.3	15.9	15	NM	LVGLS was associated (HR = 1.28 per 1% decrease) RVLS was unclear	NM	[11]
Kim *et al.*	34	NM	NM	LVGLS RVTWGLS RVFWGLS	0	0	2.5	2.5	NM	LVGLS was associated with severity (OR = 1.99 per 1% increase) RVTWGLS and RVFWGLS were not associated with severity	[12]
Janus *et al.*	31	64	NM	LVGLS	NM	NM	NM	NM	LVGLS was associated (HR = 1.52 per 1% increase)	NM	[13]
Zhang *et al.*	128	61.3 ± 13.1	67.7	RVFWLS	14.1^†^	NM	5.5	0.8	RVFWLS was associated	RVFWLS was associated with severity	[14]
Xie *et al.*	132	61 ± 13	51.5	LVGLS RV FWLS	NM	14.4	3.8	0.8	LVGLS (HR = 1.41) and RVFWLS (HR = 1.29) were associated	LVGLS and RVFWLS were associated with severity	[15]
Tryfou *et al.*	100	47.2	51	LVGLS RVGLS	NM	NM	0	NM	NM	LVGLS and RVGLS were associated with hospitalization	[16]
Temperikidis *et al.*	9	61.6	77.8	RVFWS	0	NM	0	0	RVFWS was associated	NM	[17]
Sun *et al.*	160	62.1 ± 13.4	51.9	LVLS RVFWLS	16.9^†^	NM	5.6	2.5	LVLS and RVFWLS were associated	NM	[18]
Stockenhuber *et al.*	34	72 ± 2.6	79	RVLS	NM	9	9	32	RVLS was associated (HR = 3.19)	RVLS was not associated with intubation	[19]
Stöbe *et al.*	18	64 ± 19.1	78	LVGLS LVGCS RVGLS	NM	11	5	39	NM	LVGLS, LVGCS, and RVGLS were not associated with severity	[20]
Skaarup *et al.*	174	68 ± 15	55	LVGLS	14^†^	NM	NM	NM	NM	LVGLS was associated with ARDS (HR = 1.18)	[21]
Sheehan *et al.*	56	62.5 ± 15.2	66.1	RVGLS LAGLS RAGLS	26.8	17.9	30.4^‡^	26.8	LAGLS, RVGLS, and RAGLS were not associated	NM	[22]
Rothschild *et al.*	100	64.3 ± 20.7	64	LVGLS LVFWLS RVFWLS RVLS	NM	NM	NM	NM	LVGLS was associated (HR = 0.8) LVFWLS, RVLS, and RVFWLS were not associated	LVFWLS was associated with intubation LVGLS, RVLS and RVFWLS were not associated with intubation	[23]
Park *et al.*	48	58 ± 16	67	LVGLS LVGCS RVGLS RVFWS	6.2	10.4	8.3^‡^	18.8	LVGLS was associated LVGCS, RVGLS, and RVFWS were not associated	NM	[24]
Minhas *et al.*	136	62	58	LVGLS	15	15	NM	NM	LVGLS was not associated	NM	[25]
Minardi *et al.*	120	NM	NM	RVFWLS	NM	NM	NM	NM	RVFWLS was associated	NM	[26]
Li *et al.*	120	61 ± 14	48	RVLS	NM	9.2	5	14.2	RVLS was associated (HR = 1.33)	NM	[27]
Baycan *et al.*	100	56	51	LVGLS RVLS	NM	0	0	NM	LVGLS (OR = 1.63) and RVLS (OR = 1.58) were associated	NM	[10]
Bagate *et al.*	67	61	82.1	LVGLS	0	NM	NM	NM	LVGLS was not associated	NM	[28]
Krishnamoorthy *et al.*	12	57	41.7	LVGLS RVFWS RVGS	NM	16.7	8.3	NM	RVGS and RVFWS were associated LVGLS was not associated	RVGS and RVFWS were associated with intubation LVGLS was not associated with intubation	[29]
Khani *et al.*	207	54.5 ± 14.8	57.5	LVGLS RVGLS	0	17.4	5.8	NM	LVGLS (OR = 0.2) and RVGLS (OR = 0.32) were associated	RVGLS (OR _ICU admission_ = 0.29, OR _intubation_ = 0.36) and LVGLS (OR _ICU admission_ = 0.35, OR _intubation_ = 0.35) were associated with ICU admission and intubation	[30]
Jain *et al.*	52	59.9	60	LVGLS RVGLS	21	17	10	NM	RVGLS and LVGLS were not associated	NM	[31]
Goerlich *et al.*	75	61.9 ± 13.5	59	BLS	9	9	NM	NM	BLS was not associated	BLS was not associated with ICU admission and intubation	[32]
Beyls *et al.*	79	NM	NM	LAS	NM	NM	16.5	8.9	NM	LAS (OR = 1.24) was associated with atrial fibrillation	[33]

† Instead of heart failure, cardiac disease was investigated in the literatures.

‡ Instead of COPD/Asthma, chronic pulmonary disease was investigated in the literatures.

ARDS: Acute respiratory distress syndrome; BLS: Basal longitudinal strain; CAD: Coronary artery disease; COPD: Chronic obstructive pulmonary disease; CKD: Chronic kidney disease; HF: Heart failure; HR: Hazard ratio; IHD: Ischemic heart disease; LVGLS: Left ventricular global longitudinal strain; LVLS: Left ventricular longitudinal strain; LVGCS: Left ventricular global circumferential strain; LVFWLS: Left ventricular free wall longitudinal strain; LAS: Left atrial strain; LAGLS: Left atrial global longitudinal strain; NM: Not mentioned; OR: Odds ratio; RVGLS: Right ventricular global longitudinal strain; RVGS: Right ventricular global strain; RVLS: Right ventricular longitudinal strain; RVFWS: Right ventricular free wall strain; RVFWLS: Right ventricular free wall longitudinal strain; RVFWGLS: Right ventricular free wall global longitudinal strain; RVTWGLS: Right ventricular total wall global longitudinal strain; RAGLS: Right atrial global longitudinal strain.

### Left ventricle

Several strains were measured in the left ventricle, namely the global longitudinal strain or left ventricular longitudinal strain (GLS/LS), free wall longitudinal strain (FWLS), global circumferential strain (GCS), and basal longitudinal strain (BLS).

The most common strain measured for the left ventricle was GLS/LS, which appeared across 15 studies. Nine articles reported a significant association among the 13 studies focused on GLS/LS and mortality rate. Any associations other than mortality rate would be unconcluded since current literature lacks sufficient data for other adverse effects (e.g., ICU admission, intubation, hospitalization).

### Right ventricle

In the right ventricle, the measured strains were as follows: (1) right ventricular longitudinal strain, or global strain, or longitudinal strain (RV-LS/GS/LS); (2) total wall global longitudinal strain (TWGLS); (3) free wall strain, free wall longitudinal strain, or free wall global longitudinal strain (FWS/ FWLS/ FWGLS); The most common of which being RV-LS/GS/LS (12-times) and FWS/FWLS/FWGLS (nine-times).

A significant correlation was observed between mortality rates and RV-LS measurements in 5 studies, whereas the other four did not show any associations. In the case of FWS, 6 of 8 articles reported a significant association with mortality rates.

### Left & right atrium

LS was measured in both left and right atrium (LA-LS, RA-LS), resulting in a correlation between left atrial longitudinal strain and atrial fibrillation. However, neither left nor right global longitudinal strains were associated with mortality.

## Discussion

Multiple factors could exacerbate cardiovascular function in critically ill COVID-19 patients, including systemic inflammatory response, hypoxemia, direct myocardial injury, myocarditis, arterial dysfunction, and pulmonary embolism [34–37]. Effective management of patients could be accomplished by using a reliable and straightforward predictor of mortality and adverse outcomes. Considering time efficiency and objective measuring methods of 2D-STE (i.e., Unlike conventional echocardiography, the operator's skills would not affect 2D-STE), this imaging modality can be used to determine the cardiac function and possible complications in a surge of critically ill patients and limited resources [35,38,39].

Patients evaluated with 2D-STE benefit from a highly sensitive quantitative approach for both global and regional myocardial function. For instance, GLS has a -20% cut-off value in identifying subjects at risk of developing cardiac events (18). 2D-STE would also allow us to detect subclinical cardiac dysfunction, as previously observed in patients with diabetes mellitus, hypertension, and nonobstructive coronary artery disease. 2D STE may ease the diagnosis of acute myocarditis when cardiac magnetic resonance (CMR) or endomyocardial biopsy (EMB) are unavailable [40–43]. Although STE has been frequently applied in follow-ups of COVID-19 patients thus far, the role of STE in predicting adverse cardiac outcomes have been undervalued [44–46]. We have concluded from this review that 2D-STE is a suitable technique for projecting the prognosis of the patients. But lack of sufficient research impedes us from discussing any correlations between 2D-STE measurements and ventilation rate, ICU admission, or infection severity. The results also show persistent regional abnormalities in the chronic phase of the COVID-19 disease, emphasizing the importance of long-term follow-ups. Nevertheless, the clinical significance of these findings is debatable, and future research is needed in this area.

This review highlights the potentially favorable role of 2D-STE in assessing the cardiovascular system in all COVID-19 patients. The current literature implies that left ventricular global longitudinal strain (LV GLS) and free wall global longitudinal strain (RV FWGLS) seem more reliable independent predictors of mortality rate, providing additional prognostic implications over conventional echocardiographic parameters of COVID-19 patients. Several limitations are applied in this new field of imaging in COVID-19 patients. First, there is a limited number of studies on pediatric patients with COVID-19 infection. In two of the literature, the only association examined was between global and regional strain measurements with the severity of multisystem inflammatory syndrome in children (MIS-C) [47–49].

Moreover, studies have demonstrated the link between co-morbidities such as heart failure, chronic kidney disease, and chronic obstructive pulmonary disease with decreasing strain values [50–55]. Because many patients in our included studies were senile and their co-morbid conditions could affect the findings' independence. Third, studies have only investigated 2D-STE among the adult population. The application of 2D-STE as a prognostic factor in pediatric patients needs further investigation.

It is worth mentioning that since STE detects subclinical myocardial dysfunction and impairment sensitively, it may also be a useful imaging modality for predicting outcomes in other multi-system inflammatory infectious diseases.

## Conclusion

We have stated that 2D-STE could be considered as an additional risk predictor of all-cause mortality in COVID-19 patients. The significant advantage of 2D-STE is being time-efficient and easy to measure by non-experts. However, the association between 2D-STE results and ICU requirements, intubation rate, hospitalization, and COVID-19 severity has been controversial, according to the present literature.

## Future perspective

Future research should consider the potential of 2D-STE in pediatric patients more carefully. Moreover, the role of atrial strains _especially left atrium_ in determining COVID-19 outcomes need further research which may lead to promising results. Third, the impact of 2D-STE on prognosing other multi-systemic inflammatory disorders and viral infections with a similar mechanism as COVID-19 may further help us to manage patients other than COVID-19 infection.

**Summary points**

- 2D-STE is a novel, highly sensitive, fast, and objective modality that may benefit the management of COVID-19 patients.
- Twenty-four articles that studied 2d-STE on each heart chamber were included in this review.
- Thirteen studies focused on the association between measurement of the strain GLS/LS of left ventricle and Mortality rate, nine of which found significant association.
- In case of the right ventricle, five studies found significant association between RV-LS measurements and mortality.
